# Corneal endothelial morphology in type 2 diabetes mellitus, a comparative study among Nepalese population

**DOI:** 10.1186/s12886-026-04654-7

**Published:** 2026-02-12

**Authors:** Bandana Aryal, Sarmila Acharya, Sanjeeta Sitaula, Pratap Karki, Meenu Chaudhary

**Affiliations:** Department of Ophthalmology, Institute of Medicine, B.P. Koirala Lions Center for Ophthalmic Studies, Tribhuvan University, Kathmandu, Nepal

**Keywords:** Central corneal thickness, Diabetes mellitus, Diabetic retinopathy, Endothelial cell density, Specular microscopy

## Abstract

**Background:**

To access and compare the corneal endothelial morphology between type two diabetic patients (T2DM) and non-diabetic controls, and to explore their associations with diabetes duration, HbA1c levels and severity of diabetic retinopathy in a Nepalese cohort as diabetes can lead to morphological changes in the corneal endothelium, yet data specific to the Nepalese population remain limited.

**Methods:**

A hospital-based, cross-sectional, comparative study was conducted involving 220 eyes (110 diabetic and 110 control). Non-contact specular microscopy (NIDEK CEM-530) was employed to access endothelial cell parameters, including endothelial cell density (ECD), coefficient of variation (CV), percentage of hexagonal cells (HEX), and central corneal thickness (CCT).

**Results:**

Diabetic corneas exhibited significantly lower ECD (2728.68 ± 216.40 vs. 2847.74 ± 194.97 cells/mm², *p* < 0.001) and HEX (64.93 ± 4.88% vs. 66.15 ± 4.09%, *p* = 0.0045), alongside higher CV (32.49 ± 4.26% vs. 31.01 ± 3.08%, *p* = 0.003) and CCT (545.63 ± 29.93 μm vs. 534.32 ± 27.29 μm, *p* = 0.004). Longer duration of diabetes (≥ 10 years) correlated with reduced ECD (*p* = 0.013). Eyes with DR demonstrated more pronounced endothelial dysfunction, (*p* < 0.05). HEX showed a negative correlation with HbA1c (*p* = 0.038).

**Conclusion:**

T2DM significantly alters corneal endothelial morphology and function, with worsening changes associated with prolonged diabetes, poor glycemic control, and advanced DR. These findings underscore the need for routine endothelial evaluation in diabetic patients to mitigate surgical risks and monitor corneal health.

## Background

Diabetes mellitus represents a significant global health challenge characterized by chronic hyperglycemia resulting from insulin deficiency or resistance to peripheral actions of insulin [1]. While much attention has been paid to its retinal complications, growing evidence suggests that diabetes affects all ocular structures, including the cornea and diabetic keratopathy is the most frequent clinical condition affecting human cornea which is potentially sight threatening. It is primarily caused by disturbance in the corneal epithelium and has gained significant clinical and research interest due to its severity [2]. The corneal endothelium, a single layer of non-regenerative and metabolically active hexagonal cells, plays a crucial role in maintaining corneal transparency through its pump function and barrier properties [3].Unlike other corneal layers, endothelial cells have limited proliferative capacity, they decline throughout life at an average rate of 0.6% per year [4] relying instead on cellular enlargement and migration to compensate for cell loss, processes that lead to polymegathism and pleomorphism. These morphological changes may compromise endothelial function, particularly in diabetic patients who already face systemic microvascular complications. Monitoring of endothelial cells can be performed clinically by using specular microscopy, which is an optical reflection microscope where a slit of light is focused on the corneal endothelial surface and specularly reflected light rays are focused on the film plane for viewing on a real-time monitor [5]. Several studies have investigated diabetic keratopathy in various populations, demonstrating reduced endothelial cell density, increased cell size variation, and altered corneal thickness [6–9]. However, the relationship between these changes and diabetes duration, glycemic control, and retinopathy severity remains incompletely understood, particularly in South Asian populations. Nepal faces a growing diabetes epidemic, yet data on corneal endothelial changes in Nepalese diabetics are lacking. This study addresses this knowledge gap while providing clinically relevant insights into the progression of diabetic keratopathy. By employing specular microscopy, we quantitatively assessed endothelial characteristics in diabetic patients and controls, correlating these findings with metabolic parameters and retinopathy status. Our results contribute to the understanding of diabetes-related corneal changes and underscore the importance of comprehensive ocular evaluation in diabetic patients, particularly those undergoing intraocular procedures where endothelial reserve is critical.

## Material and method

This hospital-based, cross-sectional comparative study received ethical approval from the Institutional Review Committee (IRC no: 165(6-11) E2) and adhered to the principles of the Declaration of Helsinki. Non-probability, purposive sampling was taken as a technique for sample collection. Sample size was determined by using the standard formula for comparing two independent means. In the study by Storr-Paulsen et al .2014 [6], the mean ECD was found to be 2578 (± 77) cells/ mm^2^ in the diabetic group and 2605 (± 66) cells/mm^2^ the in control group. Thus, sample size based on outcome variable was,

$${\rm{Sample size }}\left( {\rm{n}} \right) = {{\left( {{\sigma _1}^2 + {\sigma _2}^2} \right){{\left( {{{Z\alpha } \mathord{\left/ {\vphantom {{Z\alpha } {2Z\beta }}} \right. \kern-\nulldelimiterspace} {2Z\beta }}} \right)}^2}} \mathord{\left/ {\vphantom {{\left( {{\sigma _1}^2 + {\sigma _2}^2} \right){{\left( {{{Z\alpha } \mathord{\left/ {\vphantom {{Z\alpha } {2Z\beta }}} \right. \kern-\nulldelimiterspace} {2Z\beta }}} \right)}^2}} {{{\rm{d}}^2}}}} \right. \kern-\nulldelimiterspace} {{{\rm{d}}^2}}}$$

$$= {{\left( {{{77}^2} + {\rm{ }}{{66}^2}} \right){{\left( {1.96 + 0.84} \right)}^2}} \mathord{\left/ {\vphantom {{\left( {{{77}^2} + {\rm{ }}{{66}^2}} \right){{\left( {1.96 + 0.84} \right)}^2}} {{{\left( {2578 - 2605} \right)}^2}}}} \right.\kern-\nulldelimiterspace} {{{\left( {2578 - 2605} \right)}^2}}}$$

$$= 110$$

Where, 𝜎_1_ = SD of diabetes group and 𝜎_2_ = SD of control group.

𝑍𝛼/2 = 1.96 (0.05 significance level), 𝑍𝛽=0.84 (80% power), d = difference in the means of 2 groups.

**Fig. 1:**
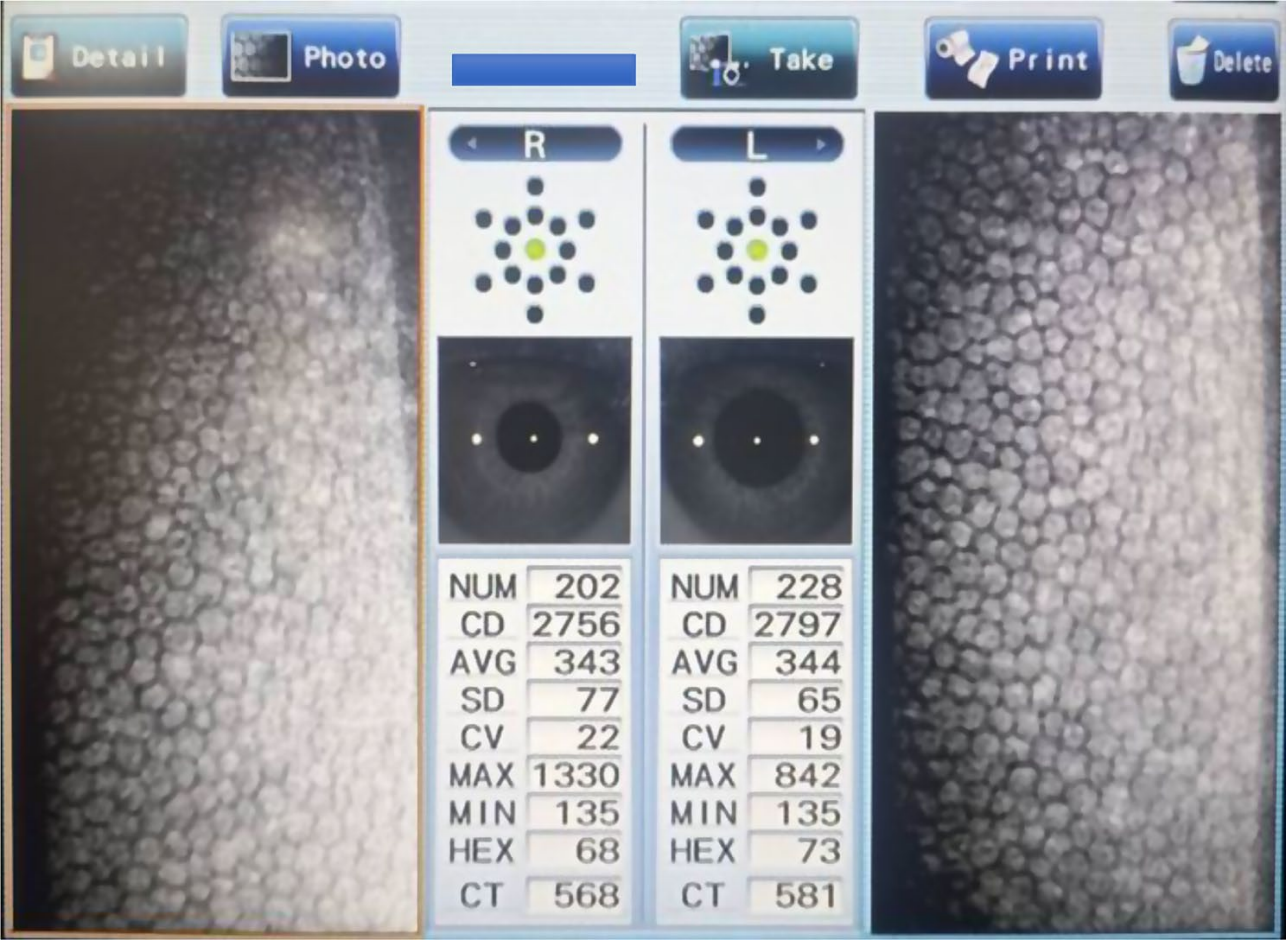
Capture result screen of NIDEK CEM-530 specular microscope showing endothelial image of a 48 y/o normal subject without diabetes

220 participants were enrolled (110 with type 2 diabetes and 110 age- and gender-matched controls) from the ophthalmology and internal medicine departments of Maharajgunj Medical Campus (Tribhuwan University Teaching Hospital) between July 2023 and April 2024. To avoid inter-eye correlation and potential selection bias, only the right eye of each participant was selected for the analysis. Diabetic participants were further stratified by disease duration (< 10 years versus ≥ 10 years), glycemic control (HbA1c < 7.5% versus ≥ 7.5%), and retinopathy status (no retinopathy, non-proliferative diabetic retinopathy, or proliferative diabetic retinopathy) based on comprehensive dilated fundus examination. To ensure sufficient statistical power and avoid small subgroup sizes, NPDR cases were analyzed as a single group rather than being classified into mild, moderate and severe stages. The diagnosis of T2DM was based on the medical history of the patient. Most recent HbA1c levels (not more than 90 days) were retrieved from laboratory reports provided by patients. All participants underwent complete ocular evaluation including visual acuity testing, anterior segment examination, intraocular pressure measurement, and detailed fundus examination. Exclusion criteria included history of corneal disease, previous intraocular/cataract surgery in the study eye (previous surgery in fellow eye were not excluded), ocular trauma, significant refractive errors, contact lens use, or any ocular/systemic condition potentially affecting corneal endothelium (e.g.: glaucoma, increased IOP, uveitis, Rheumatoid arthritis, Systemic lupus erythematosus).Corneal endothelial parameters were assessed using a NIDEK CEM-530 non-contact specular microscope following standardized protocols. The subjects were positioned on a chair in front of the specular microscope with his/her chin placed in the chin rest and forehead rested on the forehead rest area. They were asked to look at the fixation target (distant target), and the auto-alignment function was used. The endothelial images were captured in a central fixation target in an auto-capture mode (result screen shown in Fig. 1). Endothelial cell density (ECD), coefficient of variation in cell size (CV), percentage of hexagonal cells (HEX), and central corneal thickness (CCT) were calculated by automatic analysis function of the microscope. The corneal thickness was calculated from the distance between the paths of the epithelial reflection and the endothelial reflection by the microscope automatically. All measurements were performed by single Optometrist under consistent environmental conditions.

### Statistical analysis

Statistical analysis was performed using SPSS version 25. After verifying data normality through Shapiro-Wilk testing and graphical methods, the means of two variables (dependent and independent) and two groups (cases and controls) were compared using independent sample t-tests and one-way ANOVA as appropriate. Pearson correlation analysis was used to examine relationships between endothelial parameters and clinical variables including diabetes duration and HbA1c levels. A p-value less than 0.05 was considered statistically significant for all analyses.

## Results

**Fig. 2:**
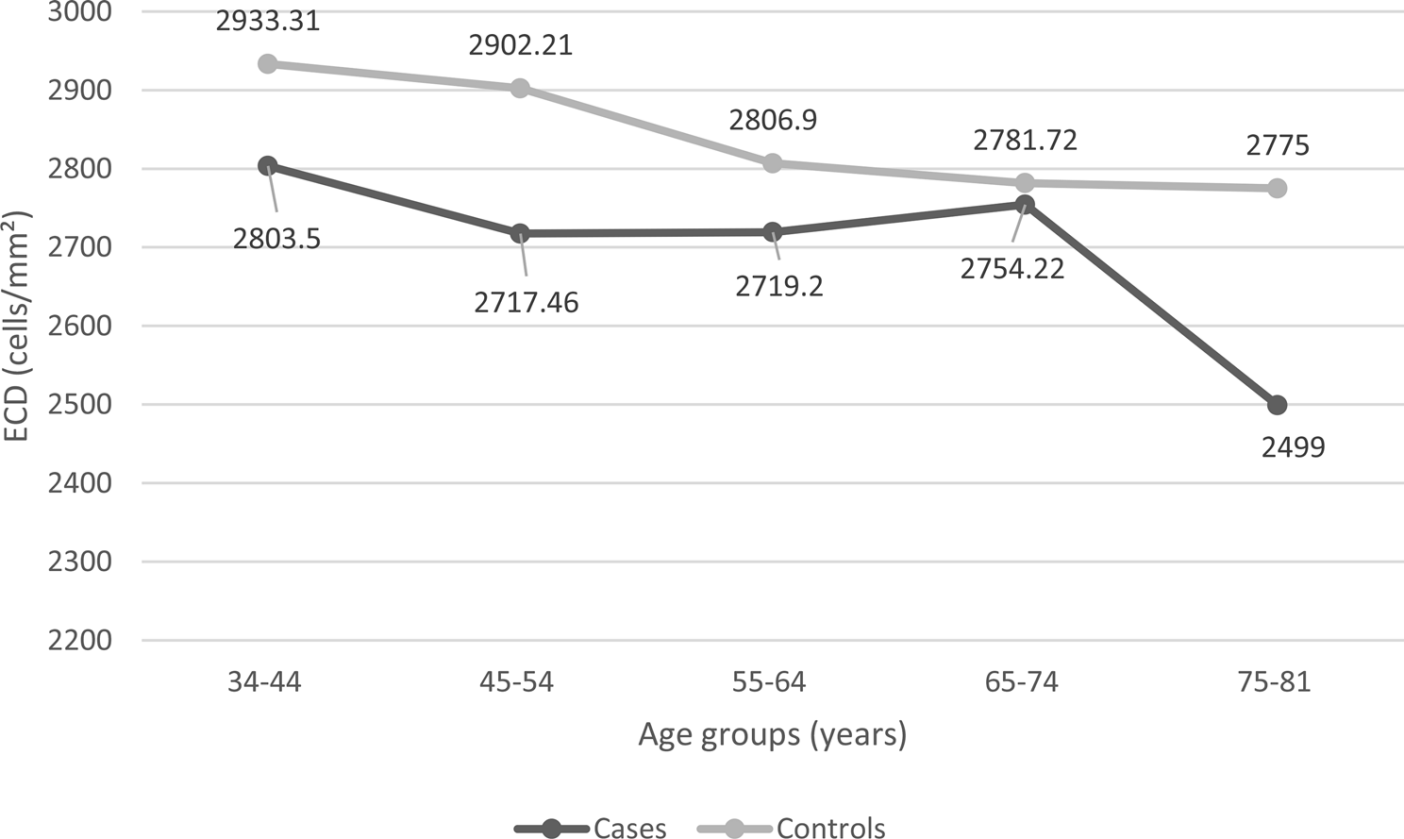
Line graph showing mean ECD among various age groups in cases and controls

A total of 220 eyes were evaluated—110 from patients with T2DM and 110 from age and gender -matched controls. Among 110, 64 were males and 46 were females. The mean age of both subjects was (54 ± 9.93) with minimum and maximum age being 34 and 81 respectively. The mean ECD was calculated among various age groups in diabetic as well as non-diabetic participants to find out the distribution of ECD. The age of all participants was divided into five groups in approximately 10-year intervals. The mean ECD among these age groups are shown in the following table (Table 1) and plotted in the following line graph (Fig. 2).

**Table 1:** ECD (cells/mm²) among various age groups in cases and controls (mean **±** SD)

Age group (year)	ECD in T2DM	ECD in Controls
34–44 (*n* = 16)	2803.50 ± 185.81	2933.31 ± 165.85
45–54 (*n* = 32)	2717.46 ± 184.02	2902.21 ± 201.40
55–64 (*n* = 40)	2719.20 ± 248.30	2806.90 ± 195.24
65–74 (*n* = 18)	2754.22 ± 209.16	2781.72 ± 179.18
75–81 (*n* = 4)	2499.00 ± 150.90	2775.00 ± 144.36

*n indicates the total number of participants in each group separately. The number of participants in each group was same for cases and controls

### I) comparision

On performing the independent sample t-test, the mean difference between ECD in T2DM and control subjects was found to be 126.78 cells/mm^2^ which was statistically significant (*p* < 0.001). ECD was significantly lower in cornea of patients with diabetes. Corneas of diabetic patients also demonstrated significantly increased CV, reduced HEX percentage and greater CCT as shown in Table 2.

**Table 2:** Comparison of endothelial cell parameters and CCT in cases and control (mean **±** SD)

Clinical data	T2DM (*n* = 110)	Control (*n* = 110)	*P* value
**ECD (cells/mm** ^**2**^ **)**	2728.68 ± 216.397	2847.74 ± 194.97	< 0.001**
**CV (%)**	32.49 ± 4.257	31.01 ± 3.075	0.003**
**HEX (%)**	64.93 ± 4.88	66.15 ± 4.09	0.0045**
**CCT (µm)**	545.63 ± 29.934	534.32 ± 27.291	0.004**

**p < 0.01

The following table (Table 3) shows a statistically significant difference in the mean of ECD between the two groups based on duration of T2DM at p value 0.013 (< 0.05). ECD was significantly lower in eyes with DM duration ≥ 10 yrs. Conversely, parameters such as CV and HEX did not exhibit any significant differences between the two groups. CCT was lower in diabetes < 10 yrs but the difference was not statistically significant.

**Table 3:** Comparison of endothelial cell parameters and CCT with duration of DM (mean **±** SD)

Duration of DM	< 10 yrs (*n* = 59)	≥ 10 yrs (*n* = 51)	*P* value
**ECD (cells/mm** ^**2**^ **)**	2776 ± 193.96	2673 ± 229.65	0.013*
**CV (%)**	32.68 ± 4.28%	32.24 ± 4.16%	0.58
**HEX (%)**	64.88 ± 4.78%	64.98 ± 5.05%	0.91
**CCT (µm)**	543.49 ± 29.17	547.51 ± 32.03	0.42

*p < 0.05

Diabetic patients with HbA1c < 7.5% had higher ECD and HEX whereas diabetic patients with HbA1c ≥ 7.50% had higher CV and CCT but none of these differences were statistically significant (*p* > 0.05) as shown in Table 4.

**Table 4:** Comparison of endothelial cell parameters and CCT with HbA1c value (mean **±** SD)

HbA1c value	< 7.50% (*n* = 62)	≥ 7.50%(*n* = 48)	*P* value
**ECD ± SD (cells/mm** ^**2**^ **)**	2738 ± 193.01	2716.85 ± 244.92	0.61
**CV ± SD (%)**	32.74 ± 4.09%	32.13 ± 4.34%	0.45
**HEX ± SD (%)**	65.39 ± 4.73%	64.33 ± 5.075%	0.26
**CCT ± SD (µm)**	544.66 ± 30.29	546.25 ± 30.96	0.95

ECD was significantly higher in eyes with no retinopathy and significantly lower in eyes with retinopathy. HEX was significantly higher in eyes with no retinopathy and significantly lower in eyes with proliferative retinopathy.CV and CCT were significantly higher in eyes with proliferative retinopathy and significantly lower in eyes without retinopathy as shown in Table 5.

**Table 5:** Comparison of endothelial cell parameters and CCT with stage of DR (mean **±** SD)

Stage of DR	No DR (*n* = 60)	NPDR (*n* = 35)	PDR (*n* = 15)	*P* value
**ECD (cells/mm** ^**2**^ **)**	2784.32 ± 194.57	2650.74 ± 235.53	2688.00 ± 199.09	0.01*
**CV (%)**	31.42 ± 3.93	33.37 ± 4.50	34.60 ± 3.22	0.009**
**HEX (%)**	66.05 ± 4.71	63.77 ± 4.62	63.13 ± 5.31	0.027*
**CCT (µm)**	539.12 ± 31.62	556.34 ± 29.04	546.67 ± 15.59	0.024*

*p < 0.05, **p < 0.01

**Fig. 3:**
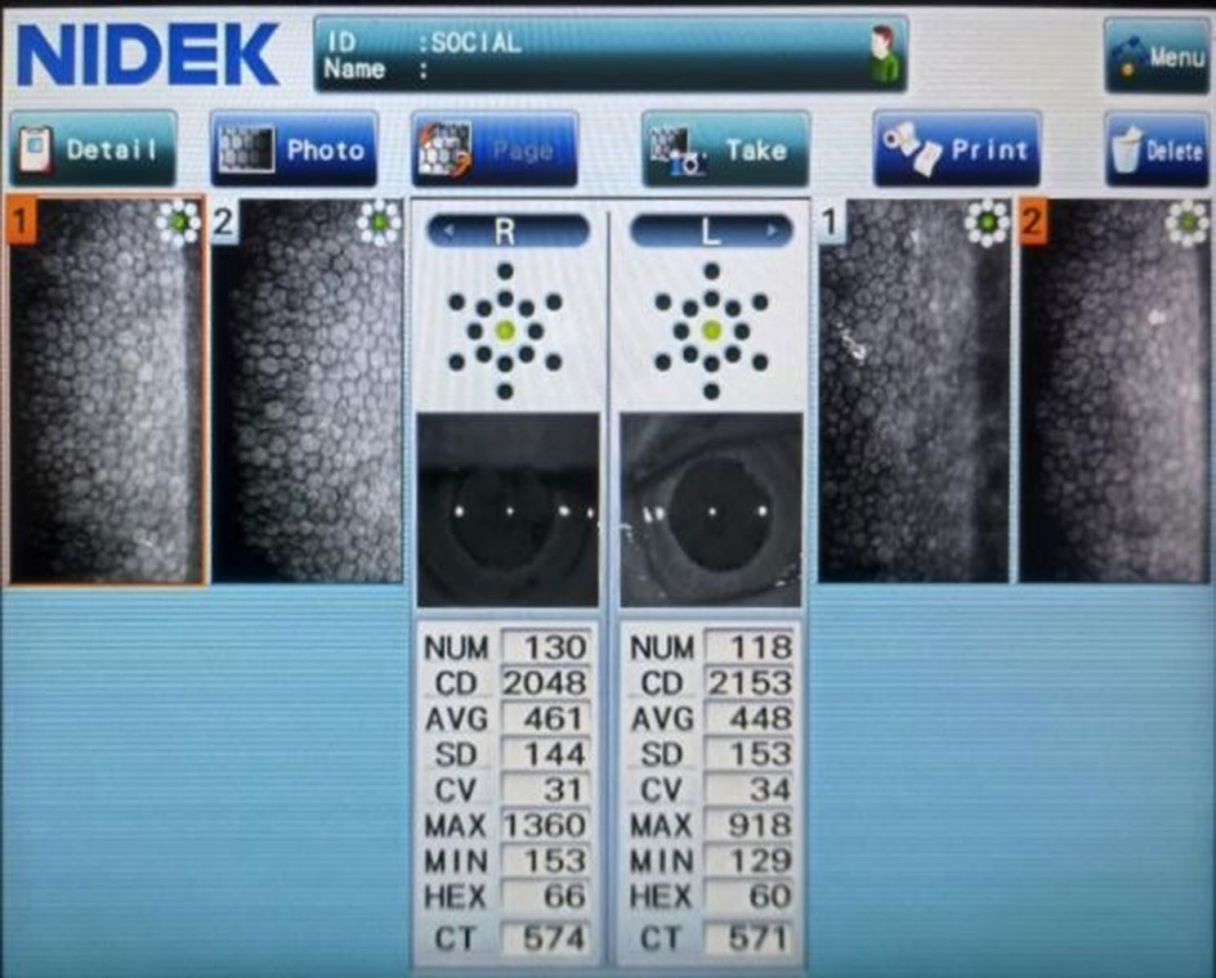
Specualr microscopy image of a 53Y/o patient with DM duration 15 yrs, 7.0% HbA1c and severe NPPDR showing low endothelial cell count and increased corneal thickness

**Fig. 4:**
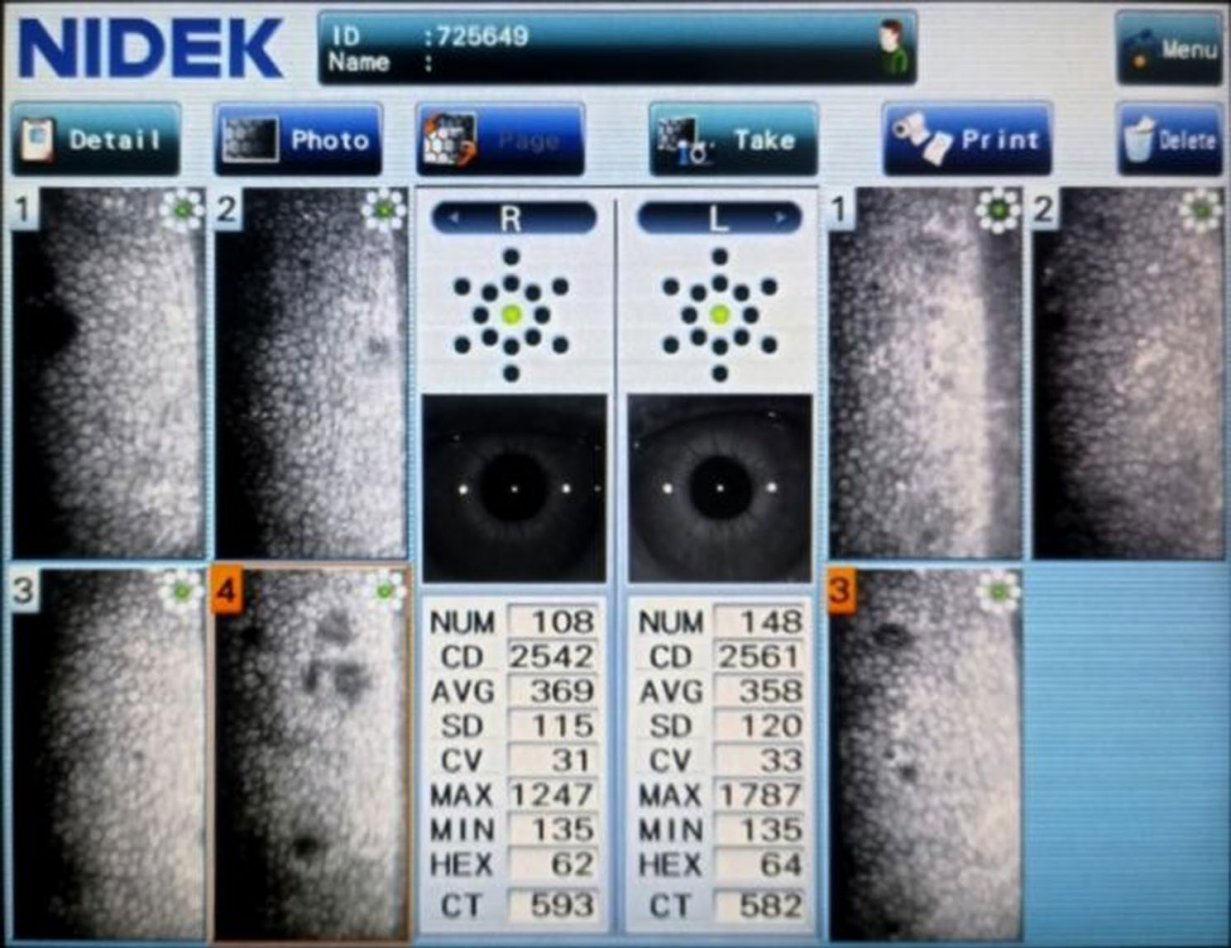
Specular microscopy image of a 60y/o patient with DM duration 12 yrs, 8.5% HbA1c and moderate NPDR showing presence of endothelial guttae along with increased CCT

**Fig. 5:**
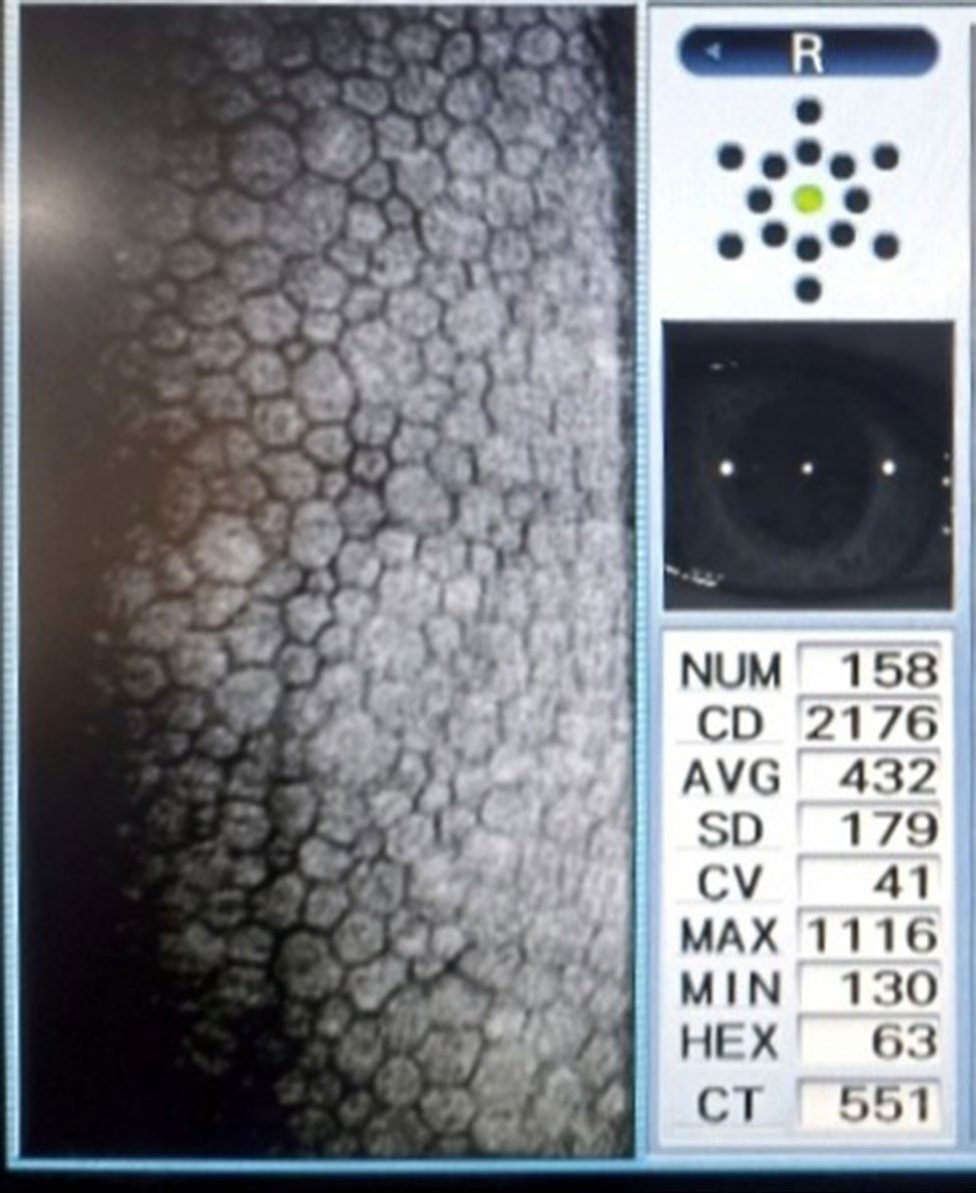
Specular microscopy image of a 48 y/0 patient with 5 yrs of diabetes and 13.1% HbA1c showing presence of polymegathism (CV > 40) along with reduced endothelial cell count

Following figures Figs. 3, 4 and 5 show specular microscopy images of patients with varying severity of diabetes, highlighting differences in endothelial cell morphology in each cases.

### II) correlation

On performing the correlation test (Table 6), ECD showed a significant negative correlation with the duration of DM i.e. as the duration of disease increases, the ECD decreases. HEX and CCT showed a positive correlation whereas CV showed a negative correlation with the duration of DM but none of these were statistically significant.

**Table 6:** Correlation among corneal morphological features and DM duration

Clinical data	Pearson correlation	*P* value
**ECD**	-0.193	0.04*
**CV**	-0.038	0.69
**HEX**	0.094	0.326
**CCT**	0.087	0.368

*Correlation is significant at p value < 0.05

Similarly, when correlated with HbA1c value, HEX had significant negative correlation with HbA1c value. Also, ECD and CCT had negative correlation with HbA1c value and CV had positive correlation with HbA1c value. However, the correlation among them was not statistically significant as shown in Table 7.

**Table 7:** Correlation among corneal morphological features and HbA1c%

Clinical Data	Pearson correlation	*P* value
**ECD**	-0.026	0.790
**CV**	0.121	0.208
**HEX**	-0.198	0.038*
**CCT**	-0.61	0.530

*Correlation is significant at p value < 0.05

## Discussion

The present study provides compelling evidence of significant corneal endothelial changes in Nepalese patients with type 2 diabetes mellitus. Our findings demonstrate that corneas in diabetics exhibit reduced endothelial cell density, increased cellular pleomorphism, and greater corneal thickness compared to non-diabetic controls, consistent with previous reports from other populations [10–13]. In our study, we found that the cornea of diabetics showed a statistically significant reduction in the mean ECD of 3.78% (*p* < 0.001) when compared to the control group. This study was similar to the results of Kim YJ et al.,2021 [12] of 1.205% significant reduction in ECD (*p* = 0.023) in Asian type 2 DM patients. Also, the results of El-Agamy et al.2017 [13] study of corneal endothelial and CCT changes in T2DM patients showed similar results of 5.24% (*p* = 0.014) significant reduction in ECD in the diabetic group. The reduction in endothelial cell density observed in our diabetic cohort, while seemingly modest, becomes clinically relevant when considering the non-regenerative nature of these cells and their critical role in maintaining corneal deturgescence. This reduction reflects cumulative damage from chronic hyperglycemia, with multiple pathogenic mechanisms potentially contributing, including advanced glycation end-product and intracellular accumulation of sorbitol, which acts as an osmotic agent leading to swelling of endothelial cells which eventually results in morphological and permeability changes in the cornea [14].

Of particular clinical importance is our observation that longer duration of diabetes correlated significantly with lower endothelial cell density (*p* = 0.04), suggesting progressive endothelial injury over time. Our result agreed with the result of Tasli NG et al., 2020 [11] who found significant negative correlation between ECD and diabetes duration (*p* = 0.038). This finding aligns with the gradual nature of diabetic microvasculature complications and emphasizes the importance of early glycemic control in preserving endothelial function. Interestingly, while poor glycemic control (HbA1c ≥ 7.5%) showed trends toward worse endothelial parameters, only hexagonality demonstrated a statistically significant negative correlation with HbA1c levels. This result was in accordance with that of Kim YJ et al. 2021 [12] who reported a significant negative correlation between HEX and HbA1c value (*p* < 0.01) .This selective effect on cellular morphology rather than density may indicate that hyperglycemia initially disrupts cell shape maintenance before progressing to cell loss.

The association between diabetic retinopathy severity and endothelial changes represents another key finding. Patients with diabetic retinopathy exhibited pronounced endothelial abnormalities, including lower cell density and hexagonality along with the highest cell size variation. This finding was in accordance with that of Jha A et al. 2022 [15] which concluded that as the DR grade worsens, the cell variation increases whereas the hexagonality decreases. This parallel deterioration of retina and cornea supports the concept of diabetes as a pan-ocular disease and suggests that retinopathy status may serve as a marker for corneal endothelial compromise. From a clinical perspective, these findings highlight the need for careful preoperative evaluation in diabetic patients, particularly those with longstanding disease or advanced retinopathy, as they may have reduced endothelial reserve and be more susceptible to surgical trauma. To minimize endothelial damage during cataract surgery and other surgeries, if necessary, endothelial protective maneuvers should be performed, and preoperative specular microscopy findings should be carefully examined in these patients.

The strength of our study is that it fills a gap in current research by offering baseline data on corneal endothelial health in Nepalese patients with diabetes. Given the increasing prevalence of diabetes in Nepal, understanding these ocular changes is very crucial. Establishing a region specific data can not only aid in risk assessment but also improve surgical outcomes and help to choose appropriate treatment strategies. This study contributes valuable region-specific insight to the global understanding of diabetes related ocular complication. The data obtained from our study can serve as reference point for future research and help guide clinical decision making in similar populations. While talking about strength, several limitations should also be acknowledged including the cross-sectional design which precludes the assessment of temporal relationships, and the potential for confounding by unmeasured variables such as medication use or systemic comorbidities. Additionally our single-center study may limit generalizability, though the inclusion of a well-characterized Nepalese population addresses an important gap in the literature. Future longitudinal studies could provide valuable insights into the progression of diabetic keratopathy and its relationship with systemic disease control.

## Conclusion

This study emphasizes that type 2 diabetes mellitus significantly affects corneal endothelial cell density and morphology in the Nepalese population, with more pronounced changes associated with longer disease duration and advanced retinopathy. These findings underscore the importance of routine corneal endothelial cell evaluation in diabetic patients, particularly those considered for intraocular surgery or with signs of retinopathy. Incorporating specular microscopy into standard diabetic eye examinations could help identify patients at risk for endothelial decompensation and guide clinical decision-making to preserve visual function.

## Data Availability

The datasets of the current study are available from the corresponding author on reasonable request.

## Abbreviations

CCT: Central Corneal thickness
CV: Coefficient of Variation
DM: Diabetes Mellitus
DR: Diabetic Retinopathy
ECD: Endothelial cell density
HbA1c: Glycated Hemoglobin
HEX: Percentage of hexagonality
NPDR: Non-proliferative diabetic retinopathy
PDR: Proliferative diabetic retinopathy
T2DM: Type II Diabetes Mellitus
